# Membranous Croup

**Published:** 1849-02

**Authors:** 


### SELECTIONS

FROM

### AMERICAN AND FOREIGN JOURNALS.

---

*Membranous Croup.*—While the article on Croup was passing through the press, we received the January No. of the "American Journal of the Medical Sciences," Art. VI. of which consists of "Extracts from the Records of the Boston Society for Medical Improvement." By Samuel Parkman, M. D., etc.

Among other things, are five cases of membranous croup treated by cauterization of the fauces and larynx with nitrate of silver, *three* of which recovered and *two* died.

What other remedial agents may have been used in the first four cases we are not informed—in the last, Dover's powder, repeated often enough to keep the patient constantly under its influence, was the only means used in addition to the caustics. R. J. B.

